# Expectant, Medical, and Surgical Management of Ovarian Endometriomas

**DOI:** 10.3390/jcm12051858

**Published:** 2023-02-26

**Authors:** Ludovico Muzii, Giulia Galati, Giulia Mattei, Alessandra Chinè, Giorgia Perniola, Violante Di Donato, Chiara Di Tucci, Innocenza Palaia

**Affiliations:** Department of Maternal and Child Health and Urological Sciences, Sapienza University of Rome, 00161 Rome, Italy

**Keywords:** endometriosis, endometrioma surgery, ovarian cysts, ovarian reserve, laparoscopy

## Abstract

Management options for ovarian endometriomas include expectant management, medical treatment, surgical treatment, in vitro fertilization (IVF), or a combination of the above. The choice of management depends on many clinical parameters that should be taken into consideration, the first of which is the main presenting symptom. Most patients are today referred to medical therapy as a first option in the case of associated pain, and to IVF in the case of associated infertility. When both symptoms are present, usually surgery is the preferred approach. Recently, however, surgical excision of an ovarian endometrioma has been associated with a postoperative reduction in the ovarian reserve, and recent guidelines suggest that the clinician should caution the patient as to the possible damage to the ovarian reserve in the case of surgery. However, evidence has been published as to a possible detrimental effect of the ovarian endometrioma on the ovarian reserve even if expectant management is followed. In this review, the current evidence on the conservative management of ovarian endometriomas, with particular focus on the issue of the ovarian reserve, is evaluated, and the different surgical techniques for the treatment of ovarian endometriomas are discussed.

## 1. Introduction

Endometriosis is a common gynecological disease with a high socio-economic impact, affecting 176 million women worldwide and approximately 10% of women of reproductive age [1]. It is a chronic, estrogen-dependent condition which is defined by the presence of endometrial glands and stroma outside the uterine cavity, mostly in the ovaries and pelvic peritoneum. Women with endometriosis may be asymptomatic or experience different types of pain, including dysmenorrhea, chronic pelvic pain or dyspareunia [2,3,4]. Endometriosis may be classified in three subtypes: superficial peritoneal endometriosis, ovarian endometrioma (OMA), and deep infiltrating endometriosis [5].

Superficial ovarian endometriosis is not amenable to a pre-surgical diagnosis, and its diagnosis is therefore made during surgery, as an incidental finding, or, at best, as a surgical confirmation of a clinical suspicion based on non-specific symptoms such as chronic pelvic pain or infertility. If found at surgery, current guidelines support surgical treatment of superficial ovarian endometriosis, both in the case of associated infertility or pain [6,7]. Both excision and ablation can be performed [8], with excision being preferable with regard to the possibility of retrieving samples for histology [6].

OMAs are present in 17–44% of young women with endometriosis [9], and may be diagnosed with high accuracy by means of transvaginal sonography [10]. Management options for OMAs include expectant management, medical treatment, surgical treatment, in vitro fertilization (IVF) in case of infertility-associated endometriosis, or a combination of the above [11]. The choice of management depends on many clinical parameters that should be taken into consideration, the first of which is the main presenting symptoms and the OMA size [6].

Surgery was considered in the recent past as the gold standard treatment in case of ovarian OMAs, in particular for cysts larger than 3 cm [12]. Recently, however, evidence has been accumulating as to a possible detrimental role of excisional surgery of the OMA on the ovarian reserve [13,14,15]. Therefore, in more recent years, a shift towards more conservative approaches to ovarian OMAs are being followed, probably with less referral to surgery, and more patients amenable to medical therapy [6]. As to surgical techniques, randomized clinical trials are being conducted in order to identify surgical techniques that may be more conservative with regard to the ovarian tissue that is left behind, and therefore more respectful of the ovarian reserve [16,17]. Additionally, some evidence has been reported as to the possibility that the presence of the ovarian OMA per se, and not only its excision, may be detrimental to the ovarian reserve [18]. As a consequence, also delaying surgery may damage the ovarian reserve. Therefore, when discussing the available management options with the patient with an ovarian OMA, the clinician is today faced with the dilemma whether to opt for surgery or to resort to medical therapy, and the issue of preserving the ovarian reserve has become pivotal in research and academic discussion. The aim of the present review is to evaluate the current evidence on the conservative management of ovarian OMAs, with particular focus on the issue of the ovarian reserve.

## 2. Ovarian Reserve in the Presence of the Endometrioma

The presence of an OMA probably influences the ovarian reserve via several mechanisms. A possible mechanism is through the compression of surrounding ovarian tissue, resulting in impaired circulation and loss of follicles. Furthermore, the inflammatory reaction in the endometriotic tissue may cause follicular damage [19]. Inflammation is related to the production of reactive oxygen species [20] that are potent inducers of tissue fibrosis, which could cause follicle loss. Therefore, it may be assumed that the continuous production of reactive oxygen species by the OMA causes a progressive decline in the ovarian reserve, probably faster than the one that occurs in healthy ovaries. In addition, inflammation may also be the cause of a reduced ovarian reserve in patients with deep endometriosis, since cytokines in the peritoneal fluid of these women correlate with a poor quality of oocytes, compared to an aged-matched control group [21,22].

Whether the decline of the ovarian reserve in women with endometriosis is progressive or not is unclear. The effects of OMAs on serum AMH levels are still controversial. It is unclear whether OMAs per se damage the ovarian reserve, or whether there is a relationship between OMA size and its effects on the ovarian reserve [23,24]. Muzii et al. found in their meta-analysis that patients with OMAs not undergoing surgery have lower AMH levels compared to patients with non-endometriotic cysts or to healthy controls, suggesting that the mere presence of OMAs may reduce the ovarian reserve [24]. Uncu et al. evaluated 30 women with an OMA and found lower AMH levels and antral follicle count (AFC) in women with an OMA compared to 30 healthy controls [25]. Chen et al., comparing patients with benign cysts and patients with OMAs, found lower AMH levels in the latter group, with no significant difference between unilateral and bilateral OMA group [26]. Additionally, Hwu et al., in a retrospective analysis, reported that the presence of OMA correlates with a lower ovarian reserve, and that bilateral OMAs cause a higher negative impact on the ovarian reserve compared to unilateral OMAs [27]. Karadag et al. reported similar results [28]. Furthermore, the latter authors found a significant negative correlation between a larger OMA size and lower AMH levels. Consequently, it is possible to conclude that the increase in the size of the OMA could be directly correlated with the damage to the ovarian reserve [28]. Very recently, however, Roman et al. reported results not consistent with the above observations. In a series of 332 patients with endometriosis, a cyst size of over 6 cm was significantly associated with higher AMH levels [29].

Only limited data are available in the literature on the evaluation of the ovarian reserve with time in patients with OMAs managed expectantly. Kasapoglu et al. concluded in their prospective study on 40 patients with ovarian OMAs and 40 healthy controls that AMH levels in women with OMAs decrease significantly faster than in healthy women. In a 6-month period, the median percentage decline in AMH levels was 26%, whereas it was 7% in healthy controls; overall, the rate of decline in serum AMH levels was not correlated with age (r = 0.22, *p* = 0.05) [18]. As yet, this is the only evidence in the literature showing a progressive decline of the ovarian reserve with expectant management. Based on this evidence, expectant management may not be as safe as is currently thought. Further confirmatory studies are needed in order to direct clinical practice.

## 3. Medical Therapy and Ovarian Reserve

What is scarcely investigated in the literature is the effect of medical therapy on the ovarian reserve in the presence of OMAs. Medical treatment options include, among others, oral contraceptives, progestins, gonadotropin-releasing hormone analogs, both agonists (GnRHa) and antagonists (GnRHant), and aromatase inhibitors (AIs). These medical treatments are based primarily on the inhibition of ovarian function, so medical or hormonal therapy should not be used in patients with associated infertility [6,7]. The only possible role of medical treatment in infertile patients is as an adjunct to IVF, in long-term GnRHs therapy before IVF, although its role has been recently questioned [6,30]. Combined oral contraceptives (COCs) and progestins are considered as first-line treatments of endometriosis, in particular when associated with pelvic pain [6]. Although COCs are one of the most frequently prescribed medical treatments for endometriosis, there is paucity of data regarding the effect of COC therapy on OMA diameter and on the ovarian reserve. In a randomized clinical trial by Harada et al., the diameter of OMAs during treatment with COCs decreased significantly more (3.0 to 2.5 mm) compared to a placebo group of patients (3.0 to 2.7 mm). No information was provided as to ovarian reserve changes during treatment [31].

Dienogest (DNG) is a fourth-generation progestin that is used in treating pain related to endometriosis [32,33,34,35,36]. DNG, thanks to its anti-inflammatory and anti-angiogenic activity, may improve reproductive outcomes in patients with endometriosis [33]. DNG is effective in reducing the size of endometriotic cysts [37,38,39,40] and has a positive effect on systemic and intra-lesional inflammatory microenvironments. In a comparative study between DNG and COCs, Angioni et al. [40] recently reported a significant reduction in OMA diameter during a 6-month treatment with DNG (from 5.4 to 3.2 cm), whereas no change was observed in the COC group. Again, no information was provided as to the change in markers of the ovarian reserve during treatment [40]. Barra et al. showed that, among patients with OMAs with a diameter of ≥4 cm, DNG significantly improves AFC and the number of oocytes retrieved during in vitro fertilization (IVF) cycles compared to a control group of patients directly undergoing IVF without medical pretreatment [33]. It is likely that, following DNG treatment, the proinflammatory environment is reduced because of the decreased size of endometriotic lesions [33]. These results are in part consistent with those reported in the paper by Muller et al., where the number of retrieved oocytes, clinical pregnancy rates, and live birth rates were significantly higher after a 6-month course of DNG administered after the excision of ovarian OMA in comparison with a control group of infertile patients undergoing IVF after surgery [41]. Contrary to these two studies, a previous study by Tamura et al., in which infertile women with stage III or IV endometriosis were treated with DNG for 12 weeks before the conventional IVF cycle, reported lower number of retrieved oocytes and lower pregnancy and live birth rates after DNG treatment compared to a control group of infertile patients not submitted to medical therapy before IVF [42].

Khalifa et al. recently reported on a series of 134 infertile patients with endometriosis randomized to a 3-month course of either GnRHa or DNG before starting controlled ovarian stimulation before IVF. The results in terms of retrieved oocytes and subsequent pregnancy rates were not different between the two groups. A significantly lower cost of treatment and fewer side effects were reported in the DNG group [43].

A recent paper by Muzoka et al. reported on a series of 49 patients randomized to a pre and post-operative 4-month course of either GnRHa or DNG. One year after the laparoscopic excision of endometriomas, more than 60% of the women randomized to DNG retained at least 70% of the pre-treatment AMH levels, whereas no patient in the GnRHa group retained the pre-treatment AMH levels (*p* < 0.01) [44].

Further studies are needed to explore the possibility that pre-treatment with DNG before controlled ovarian hyperstimulation may improve the results of the IVF cycle.

Currently, the only available study which evaluates the ovarian reserve under DNG treatment is a prospective study by Muzii et al. in which the effect of medical treatment with DNG for 6 months was evaluated in a cohort of 32 patient with unilateral OMAs. The patients treated with DNG showed a significant reduction in OMA diameter and associated pain, whereas the ovarian reserve appeared to be preserved, with a significant improvement in AFC and no significant change in AMH at the end of the six-month treatment [39]. DNG may therefore be a safe option to preserve the ovarian reserve while attempting the delay of surgery. Further studies are however needed to confirm the findings by Muzii et al. [39].

GnRHa act primarily by reducing the impact of endometriotic lesions inhibiting secretion of FSH and creating a hypoestrogenic state [45]. Therefore, GnRHa treatment could reverse a hostile proinflammatory peritoneal environment, reducing the oxidative stress and other deleterious effects associated with endometriosis, such as poor folliculogenesis and altered endometrial receptivity [45,46]. However, GnRHa have not consistently proven to be effective in reducing OMA size [47,48]. In a study by Tsujioka et al. [47], a reduction from 66.0 ± 2.0 mm to 51.0 ± 2.4 mm in cyst diameter was reported in 34 patients after a 3- to 6-month treatment with GnRHa. In contrast with the above study, Cantor et al. [48] reported no significant change in both cyst diameter (3.4 ± 0.7 to 3.2 ± 0.8 cm) and AFC (6.7 ± 2.4 to 6.4 ± 2.5) in 54 patients undergoing 2-month treatment with GnRHa. In the same study, a significant reduction in cyst diameter, and a significant increase in AFC, was reported in 50 patients undergoing a combined therapy of GnRHa and letrozole [48]. In a prospective study by Marschalek et al., GnRHa were administered every 4 weeks for 3 months in women with endometriosis and infertility. An interesting finding was that the levels of AMH and FSH levels remained stable during the administration of GnRHa [49]. This agrees with previous studies which showed that long-term treatment with GnRHa did not reduce the ovarian reserve or the function of granulosa cells of primordial or primary follicles [50].

Recently, a new class of medical drugs, i.e., GnRHant, has been introduced for the treatment of endometriosis. The efficacy of 6-month treatment with the GnRHant elagolix has been proven in two randomized clinical trial [51]. Over 70% of women randomized to 200mg of elagolix twice daily reported a significant reduction in dysmenorrhea, and over 50% of non-menstrual pelvic pain [51]. Consistent results have been reported for the GnRHant relugolix. In a randomized clinical trial, 40 mg of relugolix administred with an add-back therapy (1 mg E2 and 0.5 mg of norethisterone acetate) for six months showed a reduction of over 70% for endometriosis-associated dysmenorrhea, and over 50% for non-menstrual pelvic pain [52]. Similar results have been reported for a third GnRHant, linzagolix [53]. In all the GnRHant studies [51,52,53], enrolled patients were patients with a previous surgical diagnosis of endometriosis, with no mention of the type of disease (OMA, DIE, superficial peritoneal, or adhesions). Additionally, no mention was made of ovarian reserve markers in any of the above studies. The addition of add-back therapy to the GnRHant allows long-term treatment with no significant decrease in bone mineral density, and hypoestrogenic side effects comparable to the control arm [52,53].

In patients with endometriosis, high levels of aromatase P450 enzyme expression have been shown in eutopic endometrial tissue as well as in ectopic endometrial implants [54]. The use of AIs to suppress the locally produced estradiol by endometriotic deposits seems a promising therapy for correcting abnormal endocrine and reproductive function in these patients [55,56,57]. In a retrospective cohort study, Cantor et al. compared treatment with depo-leuprolide acetate with and without letrozole for 60 days before IVF in women with OMAs. In women treated with the addition of letrozole, AFC was higher. Additionally, the OMA diameter was significantly decreased. It may be plausible that the presence of the OMA compresses the ovarian tissue, inactivating follicular recruitment. These women showed more mature oocytes collected and a larger number of blastocysts created [48].

Scarce data are therefore present in the literature as to the effect of medical therapy on the ovarian reserve in the presence of OMAs. However, thanks to the reassuring, although limited data on medical treatment and the ovarian reserve published so far, medical treatment, particularly with DNG, may be considered as a possible treatment in the case of asymptomatic ovarian OMAs, as it seems to preserve the ovarian reserve. This treatment may even be envisaged as a preventive strategy to preserve the ovarian reserve in the presence of an unoperated OMA [39].

## 4. Surgery and Ovarian Reserve

The European Society of Human Reproduction and Embryology (ESHRE) guidelines suggest medical therapy as first-line treatment of endometriosis-associated pain [6]. Surgery is indicated in the case of non-response to medical treatment and in the case of women with infertility when a difficult access to follicles during oocyte pick up (OPU) is predicted [6]. In the ESHRE guidelines published in 2022, there is no indication on mandatory surgery with respect to cyst diameter [6]. Recently Muzii et al. presented a multiparametric score for the indication to surgery in the case of OMAs [11]. Surgery, to obtain tissue for histology diagnosis and exclude an ovarian malignancy, should be considered in case of non-reassuring sonographic features, or fast-growing cysts. Sometimes, even in absence of these suspicious scenarios, surgery should be considered, because final histology may reveal an unexpected malignancy in approximately 0.9% of cases [58].

Surgical treatment of endometriotic cysts consists of different techniques such as cystectomy, electrocoagulation, laser ablation, plasma-energy ablation and combined techniques. ESHRE guidelines suggest that surgical excision with the stripping technique is the recommended treatment for ovarian OMA [6]. The stripping technique is associated with lower cyst recurrence rates and endometriosis-associated pain recurrence, and it is related with a better post-surgical fecundability [6,59,60,61]. However, concerns have recently been raised that in some cases, the stripping technique may lead to the inadvertent removal of healthy ovarian tissue [62], which is also associated with the coagulation on the residual ovary and, consequentially, with damage to the ovarian reserve [13,15]. A recent paper reports that the decline in the ovarian reserve after surgery is not directly correlated with the amount of the ovarian tissue inadvertently removed together with the endometrioma wall during excisional surgery [63], giving indirect proof for a change not directly related to the excisional technique itself.

The recent literature shows how the surgical removal of OMAs may worsen ovarian reserve, compromising blood flow to the ovary via excessive bipolar coagulation, even when surgery is performed by skilled surgeons [64]. Ata et al. published a meta-analysis comparing the different methods used to achieve hemostasis following laparoscopic OMA excision. The decline in serum AMH levels was significantly lower with alternative hemostatic methods than with bipolar coagulation [65].

Other authors, instead, are supporters of non-excisional techniques, where the OMA wall is not removed but left in place and fenestrated, drained, and washed out, and the cyst wall is ablated with bipolar coagulation, plasma energy, or vaporized with CO_2_ laser [15,66,67]. According to these authors, ablative techniques may represent a less aggressive approach to preserve the healthy ovarian cortex, since the energy employed avoids thermal diffusion to the surrounding ovarian tissue.

Mircea et al. evaluated in a multicentric case–control study on the probability of postoperative pregnancy in patients with ovarian OMAs larger than 3 cm in diameter, managed by either ablation using plasma energy, or cystectomy [66]. They demonstrated that OMA ablation using plasma energy is a safe alternative to cystectomy, allowing high pregnancy rates in a series of infertile women with severe endometriosis, with the probability of pregnancy at 24 and 36 months after surgery in plasma energy and cystectomy groups, respectively, of 61.3% (95% CI 48.2–74.4%) vs. 69.3% (95% CI 54.5–83%) and 84.4% (95% CI 72–93.4%) vs. 78.3% (95% CI 63.8–90%) (*p* = 0.82) [67].

Candiani et al. introduced ablation using CO_2_ fiber laser technology as an easy-to-use non-excisional technique, while reporting encouraging results in terms of ovarian reserve preservation, even when performed by less experienced surgeons [68].

The same group investigated the postoperative likelihood of conception in women undergoing surgery comparing cystectomy vs. one-step CO_2_ vaporization for symptomatic OMAs. Pregnancies were recorded in 72.2% of women treated with cystectomy and in 74.3% of those managed with a CO_2_ laser; the incidence of pregnancies after ART was higher, although not significantly, in the group treated with the laser (Cystectomy: n = 6, 16.7%; CO_2_ laser: n = 15, 38.5%; *p* = 0.08). In addition, the AFC and the serum AMH levels were significantly higher after laser vaporization when compared with a cystectomy (*p* = 0.039) [68].

A recent meta-analysis by Zhang et al. reported that both cystectomy and ablation with laser or electrocoagulation were associated with a significant reduction in the ovarian reserve as evaluated with AMH. When using AFC, ablation techniques were demonstrated to be more respectful of the ovarian reserve [15]. Additionally, on this issue, further studies are needed to better evaluate which technique creates less damage to the ovarian reserve, while yielding at least comparable results in terms of disease recurrence.

Ultrasound (US)-guided aspiration and sclerotherapy are beginning to be used in the management of OMAs. Martinez Garcia et al. compared the effects of US-guided aspiration and ethanol sclerotherapy with those of laparoscopic cystectomy on AMH levels and the ovarian reserve in women with ovarian OMAs. 17-β-estradiol concentrations were significantly increased after alcohol sclerotherapy (*p* < 0.001). AFC recovery after 6 months seemed to be higher after sclerotherapy than after surgery [69]. An additional study conducted by Alborzi et al. compared assisted reproductive technique (ART) outcomes and the recurrence rate of OMAs in infertile patients undergoing sclerotherapy vs. laparoscopic ovarian cystectomy. The recurrence rates were 14.0% and 34.1% in the surgery and sclerotherapy groups (*p* = 0.017) [70]. This less aggressive approach may preserve ovarian reserve through the respect of healthy ovarian tissue, but with the possibility of higher endometriosis recurrence rates.

Recently, a new surgical technique has been proposed with the aim of combining the radical disease removal by excisional techniques, with the more conservative ovarian tissue preservation by ablation techniques. This combined technique consists of a first excisional step by cyst stripping of part of the cyst wall, followed by a second step consisting of ablation of the remaining cyst capsule in proximity of the hilus with bipolar coagulation [71], or vaporization with CO_2_ laser [72]. Promising results of this technique have been reported, and an RCT recently confirmed the non-inferiority of the combined technique compared with the stripping technique in terms of recurrences and preservation of the ovarian reserve [17].

Ovarian reserve testing has been utilized to evaluate the damage of surgery for OMAs on healthy ovarian tissue. AMH and AFC may be considered the most sensitive markers after OMA surgery, though interpretation of the results should be made with caution. As to AFC versus AMH as markers of the ovarian reserve, both have been proven to be accurate and reliable [73]. In the context of ovarian reserve evaluation in the presence of an ovarian endometrioma, AFC may represent a better marker, both for surgical or medical treatment, since it relates to the single ovary, be it either the ovary with the cyst or the contralateral healthy ovary, whereas AMH is a systematic marker of the ovarian reserve and does not control for laterality of the disease. As to the evaluation of the ovarian reserve after surgery, two systematic reviews reported consistent evidence of a 38% reduction of the ovarian reserve evaluated with AMH after excision of the OMA with the stripping technique [13,74]. Consequently, the ESHRE guideline “recommends that the clinician counsels the patient with an ovarian OMA regarding the risks of a reduced ovarian function after surgery” [6].

This scenario may be less severe if the ovarian reserve is evaluated using AFC. The post-operative AFC value has been evaluated via a meta-analysis and systematic review, which revealed that surgery for OMAs does not significantly affect the ovarian reserve [75]. A lower AFC is present on the affected ovary both before and after surgery, as proven by two prospective studies on women operated on with the combined technique for OMAs. No significant differences were found between the operated and the healthy ovary [71,72], neither for AFC or for relapse of the disease. Even in case of bilateral OMAs, Muzii et al. demonstrated that AFC was similar between the ovary operated on with the stripping technique and the contralateral ovary operated on with the combined technique [17]. Damage to the ovarian reserve is more severe in women treated for bilateral OMAs compared to unilateral procedures [76,77]. A recent study demonstrated that AMH levels were significantly (*p* < 0.05) lower in bilateral groups than in unilateral groups at the early (1 week to 1 month), intermediate (6 weeks to 6 months) and late (9–12 months) terms following surgery [78]. Whether the postoperative decline in AMH is permanent has been questioned. AMH levels reached a nadir 1 week after cystectomy, but recovered after three months. Other studies reported that the serum AMH level three months after surgery was still lower than the pre-operative levels [79,80]. In a recent meta-analysis, bilateral ovarian OMA surgery was implicated in more ovarian reserve damage as compared to a unilateral cystectomy, and the reduction of AMH levels does not appear to stop after 6 months, but decreases even further [81]. Recently Moreno-Sepulveda et al. described the deleterious effect of endometrioma surgery on short (no later than a month), medium (one and six months), and long-term (six or more months) post-operative AMH levels, in particular in the case of bilateral endometriomas and endometriomas larger than 7 cm [82]. Data in the recent literature reported a decline until 12 months, contrary to what some authors had speculated about temporary damage. Therefore, further studies evaluating ovarian reserve with a follow-up extending beyond 12 months after surgery are needed.

The surgical management of patients with endometriotic cysts is a complex procedure that requires adequate competence and skill, and dedicated operators. Data on the effect of patient age and cyst size on the decline of AMH levels at the time of surgery are not consistent in the current literature. The type of technique, laterality of OMAs (bilateral vs. unilateral), preoperative AMH level, and the hemostatic method used are considered as the main factors involved in decline of the ovarian reserve after surgery. Additional randomized trials are needed since the heterogeneity between studies is high, including different surgical methods, lengths of follow-up, and methods of ovarian reserve testing. Women should be also informed on the potential benefits and harms of surgery before IVF. In fact, there is no robust background to draw firm recommendations for systematic OMA surgery before IVF. In particular the available evidence about the risks of conservative management does not support systematic surgery before IVF in women with small OMAs [83]. Hamdan et al. investigated the effect of conservative management on the success of IVF/ICSI [84]. Women with OMAs undergoing IVF/ICSI had higher cancellation rates, but similar reproductive outcomes compared with those without endometriosis. Patients with OMAs surgically treated before IVF/ICSI, had similar reproductive outcomes in terms of LBR (OR 0.90; 95% CI [0.63, 1.28], CPR (OR 0.97; 95% CI [0.78, 1.20] and the mean number of oocytes retrieved (SMD 20.17; 95% CI [20.38, 0.05]) compared to women with untreated OMAs. A meta-analysis conducted by Nickkho–Amiry et al. came to similar conclusions, with no significant differences in pregnancy rate per cycle, clinical pregnancy rate, or live birth rate between women who underwent surgery for OMA and those who did not [85]. On the basis of this evidence, the treatment options must be discussed and individualized for each patient considering the clinical situation before IVF. Due to the lack of robust scientific evidence in the context of endometriosis-associated infertility, additional RCTs are strongly needed [86].

## 5. Conclusions

Ovarian OMAs may be managed conservatively, either with surgery respectful of the healthy ovarian tissue, or with nonsurgical alternatives such as expectant management or medical therapy.

Expectant management may be performed in case of asymptomatic OMAs, even if some authors report the possibility of a progressive decline in the ovarian reserve when the OMA is left untreated. When expectant management is decided upon, it seems wise to evaluate with serial measurements both the cyst size and the ovarian reserve, either with AMH or AFC.

Medical therapy should be considered the management of choice when the OMA is associated with pain symptoms and the imaging studies are reassuring. Among the available hormonal treatments, progestagen DNG is the drug that has been studied more extensively in recent years, with some evidence on the preservation of the ovarian reserve during medical treatment.

When surgery is indicated, the stripping technique should still be considered as the procedure of choice, since it is associated with lower recurrence rates compared to ablative techniques. New surgical techniques, such as the CO_2_ fiber laser or the plasma energy technology, still await more solid confirmatory data from the literature, and may be viewed as possible tissue-sparing alternatives to the stripping technique.

Further studies are needed to better clarify the issue of the ovarian reserve with conservative management of the OMA, particularly with medical or expectant management, where data in the literature are scanty.

## Data Availability

All data involved in this study will be made available by the corresponding author upon request.
